# A ciliated vulvar cyst mimicking a blue nevus^؆^

**DOI:** 10.1016/j.abd.2026.501431

**Published:** 2026-08-07

**Authors:** Sofía Villagrán-Essmann, Valentina Ross-Massanes, Miguel Villaseca-Hernández, Cristian Navarrete-Dechent, Leonel Hidalgo

**Affiliations:** a Department of Dermatology, School of Medicine, Pontificia Universidad Católica de Chile, Santiago, Chile; b Red de Salud UC CHRISTUS, Santiago, Chile; c Department of Pathology, School of Medicine, Pontificia Universidad Católica de Chile, Santiago, Chile; d Department of Pathology, Faculty of Medicine, Universidad de la Frontera, Temuco, Chile; e Melanoma and Skin Cancer Unit, School of Medicine, Pontificia Universidad Católica de Chile, Santiago, Chile

Dear Editor,

Pigmented vulvar lesions are relatively common, with an estimated lifetime prevalence of approximately 10% among women.^1^ A wide spectrum of entities may clinically manifest with vulvar pigmentation, including benign conditions such as postinflammatory hyperpigmentation, seborrheic keratosis, melanocytic nevi, and angiokeratomas; infectious causes such as condyloma acuminatum; and malignant neoplasms, including melanoma, basal cell carcinoma and vulvar intraepithelial neoplasia, among others.^2^ Although histopathologic examination usually allows clear differentiation among these entities, their clinical appearance often overlaps, posing a diagnostic challenge. This overlap frequently requires histopathology to establish an accurate diagnosis and rule out malignancy.

We present the case of a 36-year-old woman with no significant past medical history who was referred to dermatologic consultation for evaluation of an asymptomatic pigmented lesion on the right labium majus, first noticed one month prior. Clinical examination revealed a solitary 3-mm bluish papule (Fig. 1A). Dermoscopy demonstrated a homogeneous blue pattern without additional distinguishing features (Fig. 1B). No other mucocutaneous lesions were identified. A blue nevus was initially considered; however, given the nonspecific clinical and dermoscopic findings, an excisional biopsy was performed. Histopathologic evaluation revealed a unilocular cystic cavity located in the dermis. On higher magnification, the cyst wall was lined by a ciliated metaplastic epithelium with no cytologic atypia, confirming the diagnosis of a ciliated cyst of the vulva (Fig. 2A–B).

**Figure 1:**
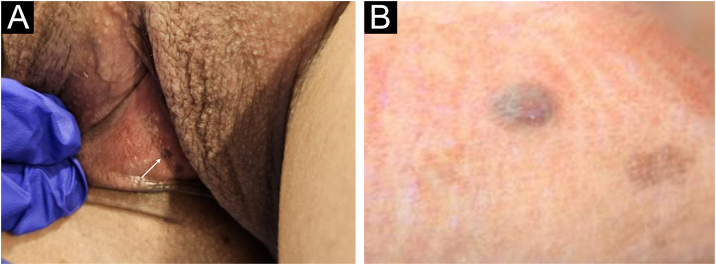
Vulvar cyst. (A) Clinical features showing a bluish papule on the right labium majus. (B) Dermoscopic image of the lesion revealing a well-defined blue papule with a homogenous pattern (non-polarized light, non-contactdermoscopy, original magnification 10×).

**Figure 2:**
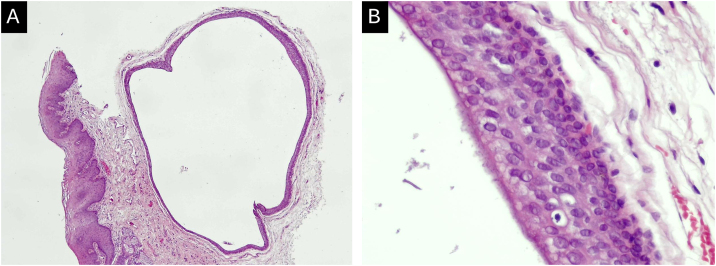
(A) Microphotograph of a ciliated cyst: An unilocular cystic cavity (Hematoxylin & eosin, ×40). (B) Higher magnification reveals a ciliated metaplastic epithelium, confirming the diagnosis of a ciliated vulvar cyst (Hematoxylin & eosin, ×100).

Ciliated cysts of the vulva are rare, benign cystic lesions. Its origins are highly debated, with proposed mechanisms including that they may derive from Müllerian or mesonephric rests, metaplasia, or cystic dilation of normal vulvar glands.^3^ Reported lesions usually present as nonpigmented cystic nodules or swellings, often involving the labia minora, vestibule, or labium majus. Most reported cases are nonpigmented and present as translucent cystic nodules.^3^, ^4^, ^5^ To the best of our knowledge, no previous cases have described a ciliated cyst  manifesting as a solitary bluish papule, clinically mimicking more common pigmented genital lesions such as blue nevus, angiokeratoma, or even condyloma acuminatum.^6^

Diagnosis is generally clinical, but complementary imaging techniques such as ultrasound or magnetic resonance imaging can be helpful in delineating the lesion’s extent and its relationship with the urethra. Treatment of symptomatic or diagnostically uncertain vulvar cysts is complete excision, which is usually curative.^4^, ^5^ In our case, excisional biopsy not only provided a definitive diagnosis but also served as adequate therapy, with no recurrence observed on follow-up.

Although rare, dermatologists should be aware of this entity, as its unusual presentation may mimic a wide range of benign and malignant vulvar lesions. Including ciliated vulvar cysts in the differential diagnosis of solitary pigmented or bluish genital papules may prevent misdiagnosis and unnecessary concern.

## ORCID ID

Sofía Villagrán-Essmann: 0009-0004-5733-9889

Miguel Villaseca-Hernández: 0000-0002-3532-5302

Cristian Navarrete-Dechent: 0000-0003-4040-3640

## Research data availability

Does not apply.

## Financial support

None declared.

## Authors’ contributions

Sofía Villagrán-Essmann: Approval of the final version of the manuscript; design and planning of the study; drafting and editing of the manuscript; collection, analysis and interpretation of data; effective participation in research orientation; intellectual participation in the propaedeutic and/or therapeutic conduct of the studied cases; critical review of the literature; critical review of the manuscript.

Valentina Ross-Massanes: Design and planning of the study; drafting and editing of the manuscript; collection, analysis and interpretation of data; effective participation in research orientation; intellectual participation in the propaedeutic and/or therapeutic conduct of the studied cases; critical review of the literature; critical review of the manuscript.

Miguel Villaseca-Hernández: Approval of the final version of the manuscript; collection, analysis and interpretation of data; intellectual participation in the propaedeutic and/or therapeutic conduct of the studied cases; critical review of the manuscript.

Cristian Navarrete-Dechent: Approval of the final version of the manuscript; drafting and editing of the manuscript; collection, analysis and interpretation of data; intellectual participation in the propaedeutic and/or therapeutic conduct of the studied cases; critical review of the manuscript.

Leonel Hidalgo: Approval of the final version of the manuscript; drafting and editing of the manuscript; collection, analysis and interpretation of data; intellectual participation in the propaedeutic and/or therapeutic conduct of the studied cases; critical review of the manuscript.

## Conflicts of interest

None declared.
